# Exploring the *Artemisia* Genus: An Insight into the Phytochemical and Multi-Biological Potential of *A. campestris* subsp. *lednicensis* (Spreng.) Greuter & Raab-Straube

**DOI:** 10.3390/plants11212874

**Published:** 2022-10-27

**Authors:** Adriana Trifan, Monika E. Czerwińska, Constantin Mardari, Gokhan Zengin, Kouadio Ibrahime Sinan, Izabela Korona-Glowniak, Krystyna Skalicka-Woźniak, Simon Vlad Luca

**Affiliations:** 1 Department of Pharmacognosy, Faculty of Pharmacy, “Grigore T. Popa” University of Medicine and Pharmacy Iasi, 700115 Iasi, Romania; 2 Department of Biochemistry and Pharmacogenomics, Faculty of Pharmacy, Medical University of Warsaw, 02-097 Warsaw, Poland; 3 Center for Preclinical Research, Medical University of Warsaw, 02-097 Warsaw, Poland; 4 Botanic Garden “Anastasie Fatu”, 700471 Iasi, Romania; 5 Physiology and Biochemistry Research Laboratory, Department of Biology, Science Faculty, Selcuk University, Konya 42130, Turkey; 6 Department of Pharmaceutical Microbiology, Faculty of Pharmacy, Medical University of Lublin, 20-093 Lublin, Poland; 7 Department of Natural Products Chemistry, Medical University of Lublin, 20-093 Lublin, Poland; 8 Biothermodynamics, TUM School of Life and Food Sciences, Technical University of Munich, 85354 Freising, Germany

**Keywords:** wormwood, LC-HRMS/MS, flavonoids, antioxidant, anti-enzymatic, pro-inflammatory cytokines

## Abstract

The *Artemisia* L. genus includes over five hundred species with great economic and medicinal properties. Our study aimed to provide a comprehensive metabolite and bioactivity profile of *Artemisia campestris* subsp. *lednicensis* (Spreng.) Greuter & Raab-Straube collected from north-eastern Romania. Liquid chromatography with tandem high-resolution mass spectrometry (LC-HRMS/MS) analysis of different polarity extracts obtained from the aerial parts led to the identification of twelve flavonoids, three phenolic acids, two sesquiterpene lactones, two fatty acids, one coumarin, and one lignan. The antioxidant and enzyme inhibitory properties were shown in the DPPH (0.71–213.68 mg TE/g) and ABTS (20.57–356.35 mg TE/g) radical scavenging, CUPRAC (38.56–311.21 mg TE/g), FRAP (121.68–202.34 mg TE/g), chelating (12.88–22.25 mg EDTAE/g), phosphomolybdenum (0.92–2.11 mmol TE/g), anti-acetylcholinesterase (0.15–3.64 mg GALAE/g), anti-butyrylcholinesterase (0–3.18 mg GALAE/g), anti-amylase (0.05–0.38 mmol ACAE/g), anti-glucosidase (0.43–2.21 mmol ACAE/g), and anti-tyrosinase (18.62–48.60 mg KAE/g) assays. At 100 μg/mL, *Artemisia* extracts downregulated the secretion of tumor necrosis factor (TNF)-α in a lipopolysaccharide (LPS)-stimulated human neutrophil model (29.05–53.08% of LPS+ control). Finally, the *Artemisia* samples showed moderate to weak activity (minimum inhibitory concentration (MIC) > 625 mg/L) against the seventeen tested microbial strains (bacteria, yeasts, and dermatophytes). Overall, our study shows that *A. campestris* subsp. *lednicensis* is a promising source of bioactives with putative use as food, pharmaceutical and cosmetic ingredients.

## 1. Introduction

The *Artemisia* L. genus (Asteraceae) comprises over five hundred perennial species inhabiting mainly the Northern Hemisphere, especially the arid and semi-arid temperate regions of Europe, North America, and Asia [1]. *Artemisia* species are small herbs or shrubs with a specific bitter taste and pungent aroma assigned to terpenoids, mostly monoterpenes in the essential oil and sesquiterpene lactones [2]. This genus has significant economic (food, spices, beverages, and ornamental use) and medicinal properties due to its chemical diversity, which includes, aside from terpenoids, other phytochemicals such as flavonoids, phenolic acids, coumarins, sterols, and lignans [2,3,4]. *Artemisia* species have a long-established traditional use, being utilized to alleviate various ailments (e.g., digestive and hepatobiliary complaints, inflammatory diseases, malaria, bronchitis, helminthiasis, and cancer) [5]. The isolation of artemisinin as the active principle of *A. annua* against malaria (1971) has attracted the interest of the scientific community toward the genus and has prompted its extensive research in drug discovery and development [6]. Thus, various *in vitro*, *in vivo*, and clinical studies have revealed its versatile pharmacological profile characterized by antimalarial, anthelmintic, antitubercular, antiviral, antiemetic, hepatoprotective, gastroprotective, antihyperlipidemic, antidiabetic, antihypertensive, anti-asthmatic, antidepressant, anxiolytic, anticancer, and insecticidal properties [3,7,8,9].

The pleiotropic pharmacology of the *Artemisia* genus resides in its vast number of species colonizing areas with different ecological conditions and types of vegetation, which translates into different morphological and biological characteristics [10,11,12,13]. Furthermore, this leads to both qualitative and quantitative variations in the phytochemical profile, consequently impacting the bioactivities of the species. Therefore, it is of great interest to further investigate the potential of *Artemisia* genus metabolites to achieve significant alleviation of various human diseases [14].

**Figure 1:**
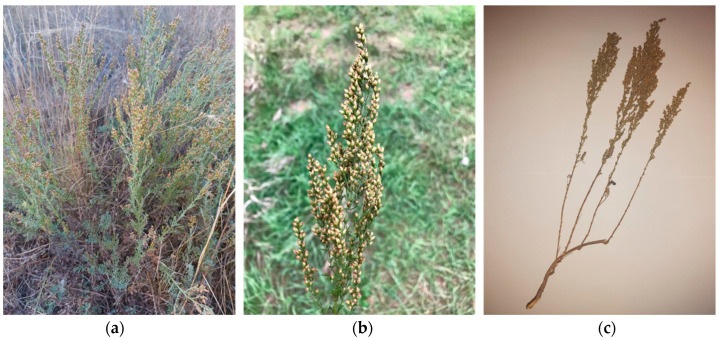
*Artemisia campestris* subsp. *lednicensis* (Spreng.) Greuter & Raab-Straube. (**a**) In its natural habitat, near Aroneanu Lake (Iasi county, Romania); (**b**) inflorescence; (**c**) herborized plant material (Photo by Adriana Trifan).

*Artemisia campestris* subsp. *lednicensis* (Spreng.) Greuter & Raab-Straube (syn. *A. lednicensis* Rochel ex Spreng.) belongs to an infraspecific taxon of *Artemisia campestris* L. [15]. This species frequently inhabits sunny meadows in steppe regions of Romania characterized by sand or loess substrate [16]. It is a perennial, more or less tomentose, and odorless plant, with ovoidal-shaped, sessile, and erect anthodia (Figure 1). *A. campestris* subsp. *lednicensis* has been used in Romanian traditional medicine as a tonic, anthelmintic, cholagogue, emmenagogue, and antiseptic agent [17]. To the best of our knowledge, no studies on the phytochemistry nor the biological attributes of *A. campestris* subsp. *lednicensis* have been undertaken.

In our endeavor to promote interest in the Romanian *Artemisia* species [18], we report herein for the first time on the metabolite and biological profile of *A. campestris* subsp. *lednicensis* aerial parts. The phytochemical characterization was assessed by means of LC-HRMS/MS. The bioactivity screening included the evaluation of (i) antioxidant effects (*in vitro* free radical scavenging, metal chelating and reducing power, and total antioxidant activity); (ii) enzyme inhibitory activity (*in vitro* anti-cholinesterase, anti-amylase, anti-glucosidase and anti-tyrosinase effects); (iii) influence upon pro-inflammatory cytokines secretion from *ex vivo* LPS-stimulated human neutrophils; and (iv) *in vitro* antimicrobial potential (against Gram-positive and Gram-negative bacteria, yeasts, and dermatophytes).

## 2. Results and Discussion

### 2.1. Total Phenolic and Flavonoid Content

In phytochemical studies, the phenolic and flavonoid content of a plant extract is usually determined to obtain the first insights into its pharmacological potential. The total content of these bioactive metabolites in the tested extracts is presented in Table 1. Apparently, the levels are dependent on the used extraction solvents. The highest concentration of the total phenolic was determined in the hydroalcoholic extract with 104.00 mg GAE/g, followed by methanol (84.42 mg GAE/g), water (71.73 mg GAE/g), dichloromethane (20.67 mg GAE/g), and hexane (17.11 mg GAE/g). Regarding the total flavonoid content, the methanol extract was the richest with a value of 23.13 mg RE/g, and the hydroalcoholic (15.08 mg RE/g) and dichloromethane (16.43 mg RE/g) extracts contained similar levels of flavonoids (*p* > 0.05). Interestingly, the water extract had the lowest total flavonoid content. From these results, we concluded that the methanolic and hydroalcoholic solvents could be utilized for the extraction of bioactive compounds from *A. campestris* subsp. *lednicensis*. Consistent with our results, several investigators have shown the efficacy of polar solvents for preparing extracts from *Artemisia* plants [19,20]. In addition, the alcohol–water mixture showed synergistic effects for the extraction of phenolics and flavonoids in several studies [21,22]. In the literature, different levels of the total phenolic and flavonoid content have been reported for the species of the genus *Artemisia* [23,24,25,26]. However, the results of spectrophotometric assays are very controversial since not only phenolic/flavonoid compounds but also other phytochemicals (peptides, sulfides, etc.) could react with the used reagents and therefore the results obtained are not entirely accurate [27]. Therefore, to confirm the spectrophotometric results, further techniques including LC-MS, LC-HRMS/MS, or LC-NMR are needed to detect the chemical constituents of plant extracts.

### 2.2. LC-HRMS/MS Metabolite Profiling

The five *A. campestris* subsp. *lednicensis* aerial part extracts achieved with solvents of different polarities were thoroughly analyzed by LC-HRMS/MS.

**Figure 2:**
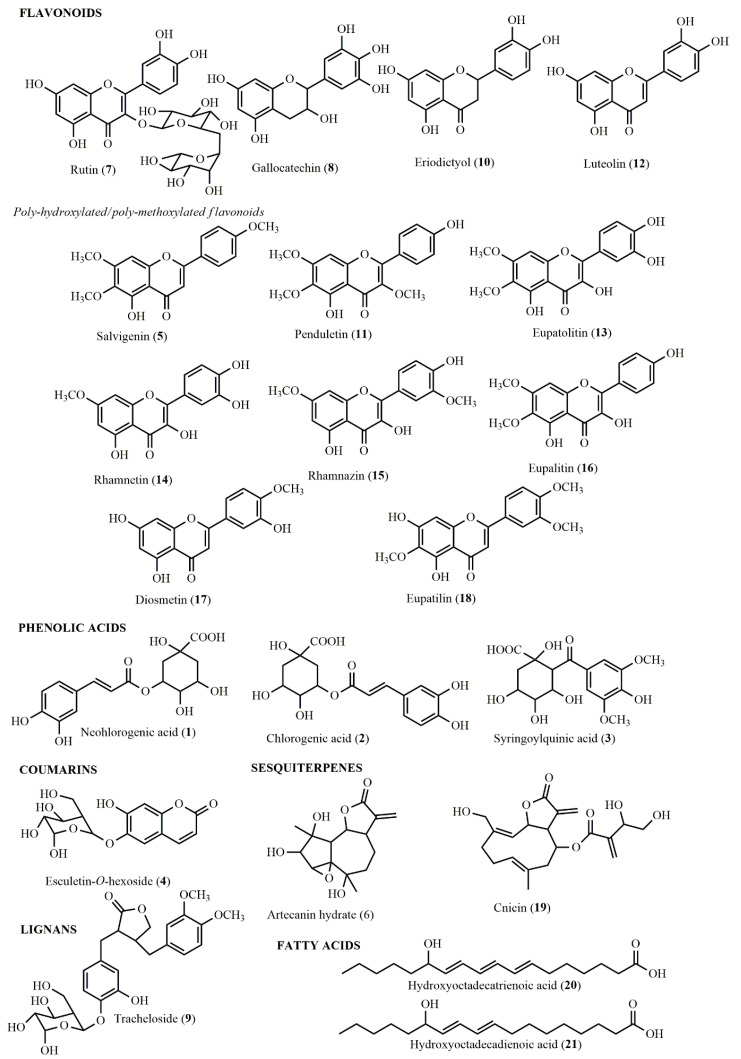
Chemical structures of the compounds tentatively labeled in the *A. campestris* subsp. *lednicensis* aerial part extracts.

The annotation of the peaks was assessed by a comparison of the spectral and chromatography data with the literature [18,28,29,30,31,32,33] and databases (KNApSAcK, METLIN, NIST, etc.). Thus, 21 specialized metabolites from various phytochemical groups (phenolic acids, coumarins, flavonoids, sesquiterpenes, fatty acids, and lignans) were fully or partly ascribed (Table 2, Figure 2).

Flavonoids were the representative category of compounds, with 12 distinct congeners spotted in the analyzed extracts. Quercetin-*O*-deoxyhexoside-*O*-heoxisde (e.g., rutin) presented the pseudomolecular ion [M–H]^–^ at *m/z* 609.1476 (C_27_H_29_O_16_^–^) and its characteristic MS/MS fragments at *m/z* 300.0287 [Quercetin–2H]^–^, 271.0255 [Quercetin–CH_2_O–H]^–^, and 151.0075 (resulted after the specific Retro-Diels Alder, RDA, cleavage of quercetin). The RDA cleavage of flavonoids has been extensively detailed by Fabre et al. [34]. These fragments were in agreement with those presented by Melguizo-Melguizo et al. [31] for the structure of rutin. Gallocatechin (**8**) presented the [M–H]^–^ ion at *m/z* 305.0671 (C_15_H_13_O_7_^–^) and its main MS/MS fragment ion at *m/z* 225.1144. Next, eriodictyol (**10**) was tentatively assigned based on its pseudomolecular ion at *m/z* 287.0567 (C_15_H_11_O_6_^–^) and RDA fragment ions at *m/z* 151.0046 and 135.0479, as proposed by Fabre et al. [34]. The presence of luteolin (**12**), [M–H]^–^ at *m/z* 285.0567, was confirmed by standard injection and diagnostic MS/MS fragments at *m/z* 175.0386 [M–C_3_O_2_–C_2_H_2_O–H]^–^ and 133.0313 (RDA fragment) [34].

The remaining flavonoids were poly-hydroxylated/poly-methoxylated flavone derivatives. Due to the high isomerism, assigning the exact position of the hydroxy and methoxy group with the use of LC-HRMS/MS only is difficult. However, for simplification purposes, some possible structures have been proposed. Peak **5** with the [M–H]^–^ ion at *m/z* 327.0847 (C_18_H_15_O_6_^–^) was suggested to have three methoxy groups, based on the RDA fragment ions at *m/z* 177.0415 (C_10_H_9_O_3_^–^) and 151.0075 (C_8_H_7_O_3_^–^) obtained after cleavage of the pyran ring [33]. Thus, its tentative structure was proposed as hydroxytrimethoxyflavone, possibly belonging to salvigenin. Two isobaric peaks (**11** and **18**) with the pseudomolecular ions [M–H]^–^ at *m/z* 343.0813 (C_18_H_15_O_7_^–^) indicated a flavone derivative with an additional hydroxy group compared to peak **5**. The MS/MS fragmentation patterns of peaks **11** and **18** showed the successive loss of three methoxy groups, as follows: 328.0382 [M–CH_3_]^–^, 313.0382 [M–2×CH_3_]^–^, and 298.013 [M–3 × CH_3_]^–^. Thus, the two dihydroxytrimethoxyflavones were putatively assigned as penduletin (**11**) and eupatilin (**18**) [29]. In the MS/MS fragmentation of compound **13** ([M–H]^–^ at *m/z* 345.0602, C_17_H_13_O_8_^–^), only two methoxy groups were indicated by the MS/MS fragment ions at *m/z* 330.0402 [M–CH_3_]^–^ and 315.0188 [M–2×CH_3_]^–^. Thus, a number of four hydroxy groups were assumed to be attached to the flavone skeleton, suggesting a tetrahydroxydimethoxyflavone, possibly belonging to eupatolitin [29]. Peak **14** with [M–H]^–^ at *m/z* 315.0509 (C_16_H_11_O_7_^–^) indicated a flavone with one methoxy group less than eupatolitin, thus a tetrahydroxymethoflavone (e.g., rhamnetin) [35]. Two isobaric peaks **15** and **16** with the pseudomolecular ions at *m/z* 329.0678 (C_17_H_13_O_7_^–^) were assigned as dihydroxydimethoxyflavones. The two methoxy groups were evidenced by the MS/MS fragment ions at *m/z* 314.0456 [M–CH_3_]^–^ and 299.0241 [M–2 × CH_3_]^–^. After the sequential loss of these two groups, the smaller fragments were characteristic to flavonoids structures, with the diagnostic RDA ions at *m/z* 151.0068 and 133.0347. In agreement with previous reports on similar compounds from the *Artemisia* genus, these two compounds were tentatively labeled as rhamnazin (**15**) and eupalitin (**16**) [29,35]. Finally, diosmetin (**17)**, [M–H]^–^ at *m/z* 299.0566) was indicated by the presence of a single methoxygroup with the characteristic MS/MS fragment ion at *m/z* 284.0259 [M–CH_3_]^–^; all the remaining fragments confirmed the flavone structure [29]. The high abundance of poly-hydroxylated/poly-methoxylated flavone is specific to *Artemisia* species and can have chemotaxonomical importance [18]. All of these phytochemicals have been previously reported in *A. annua*, *A. austriaca*, *A. pontica*, *A. vulgaris*, or *A. absinthium* [18].

Regarding the phenolic acids from *A. lednicensis*, it is worth mentioning the presence of quinic acid derivatives such as neochlorogenic (**1**) and chlorogenic acid (**2**) as well as syringoylquinic acid (**3**). Confirmed by standard injection, the two caffeoylquinic isomers were also indicated by their characteristic fragmentation patterns. While neochlorogenic acid (**1**) presented the main MS/MS fragment ions at *m/z* 191.0484 [Quinic acid–H]^–^, 179.0252 [Caffeic acid–H]^–^ and 135.0370 [Caffeic acid–CO_2_–H]^–^, chlorogenic acid (**2**) showed diagnostic fragments at *m/z* 191.0568 [Quinic acid–H]^–^, 173.0252 [Quinic acid–H_2_O–H]^–^, and 135.0370 [Caffeic acid–CO_2_–H]^–^. These differences in the fragmentation patterns have also been reported in previous literature [36].

Esculetin-*O*-hexoside (**4**) was the sole coumarin putatively identified in some of the extracts of *A. lednicensis*. The pseudomolecular ion at *m/z* 339.0715 (C_15_H_15_O_9_^–^) generated the MS/MS fragments at *m/z* 177.0233 [Esculetin–H]^–^, 149.0157 [Esculetin–CO–H]^–^, and 133.0217 [Esculetin–CO_2_–H]^–^, in agreement with Olennikov et al. [29].

In addition, one glycosylated lignan, possibly belonging to the structure of tracheloside (**9**), was labeled. This lignan was also previously present in *A. annua*, *A. austriaca*, and *A. vulgaris* [18]. Finally, two sesquiterpenes, artecanin hydrate (**6**) and cnicin (**19**), and two fatty acids, hydroxyoctadecatrienoic acid (**20**) and hydroxyoctadecadienoic acid (**21**), were tentatively annotated. These classes of specialized metabolites were evidenced in *A. annua*, *A. pontica*, *A. austriaca*, *A. vulgaris*, or *A. absinthium* [18].

**Figure 3:**
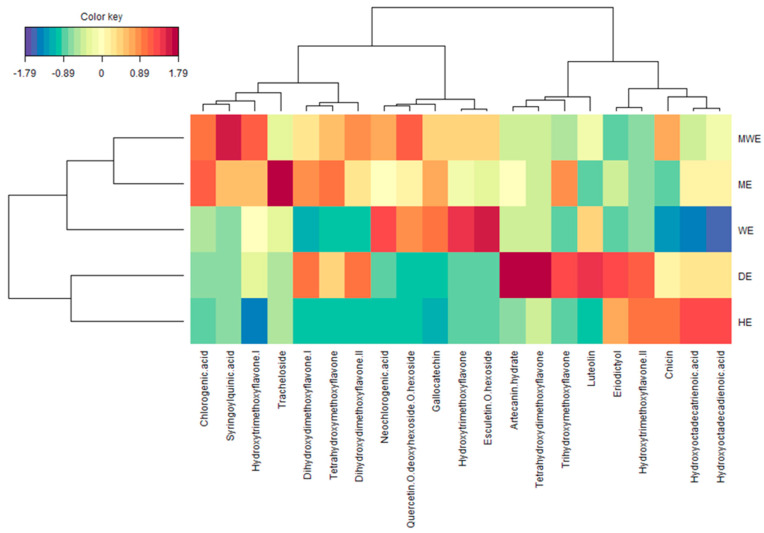
Clustered image map (red color: high content; blue color: low content) on the phytochemical dataset of the *A. campestris* subsp. *lednicensis* aerial part extracts.

To obtain a better view of the relative concentration of phytochemicals present in each solvent, we then performed a thorough clustered image map analysis using peak area data (Figure 3). For example, the hexane extract contained a high level of eupatilin (**18**). Rhamentin (**14**) is the main molecule of the dichloromethane extract, but important amounts of eupatilin (**18**), eriodictyol (**10**), and artecanin hydrate (**6**) were also determined. Both hydroalcoholic and methanol extracts contained relatively high levels of syringoylquinic acid (**3**).

### 2.3. Antioxidant Properties

The antioxidant activities of plants are an important marker to evaluate their pharmacological properties. This fact could provide valuable information on their defense abilities against free radical attacks. Because the term antioxidant reflects a broad spectrum of chemical effects, a singular assay does not suffice to unveil the antioxidant picture of a plant extract. Therefore, various tests with different mechanisms must be performed in phytochemical studies. With this in mind, we performed several assays to determine the antioxidant properties of *Artemisia campestris* subsp. *lednicensis* extracts and the results are shown in Table 3. These assays were classified according to the radical scavenging (ABTS and DPPH), reducing power (FRAP, CUPRAC, and PBD), and metal chelating effects. In both radical scavenging assays, the highest activity was found for the methanol extract (DPPH: 213.68 mg TE/g; ABTS: 356.35 mg TE/g), followed by the hydroalcoholic, water, dichloromethane, and hexane extracts. However, the ABTS scavenging abilities of the hydroalcoholic and water extracts did not differ statistically (*p* > 0.05). The electron-donation abilities of antioxidant compounds, namely reducing power, is one of the most relevant markers in their antioxidant mechanism. Thus, we tested the conversion of Cu^2+^ to Cu^+^ in the CUPRAC assay in the presence of antioxidant compounds as well as Fe^3+^ to Fe^2+^ in the FRAP assay. In the reducing power assays, the most active extract was methanol with values of 311.21 mg TE/g (in CUPRAC assay) and 202.34 mg TE/g (in FRAP assay). In both reducing power assays, the hexane extract displayed the weakest abilities with values of 38.56 mg TE/g in the CUPRAC and 21.68 mg TE/g in the FRAP assay, respectively. As presented in Table 3, in both radical scavenging and reducing power assays, the *Artemisia* extracts showed a similar decreasing pattern of activity. Therefore, we deduced that the same compounds might play a role in these assays.

**Figure 4:**
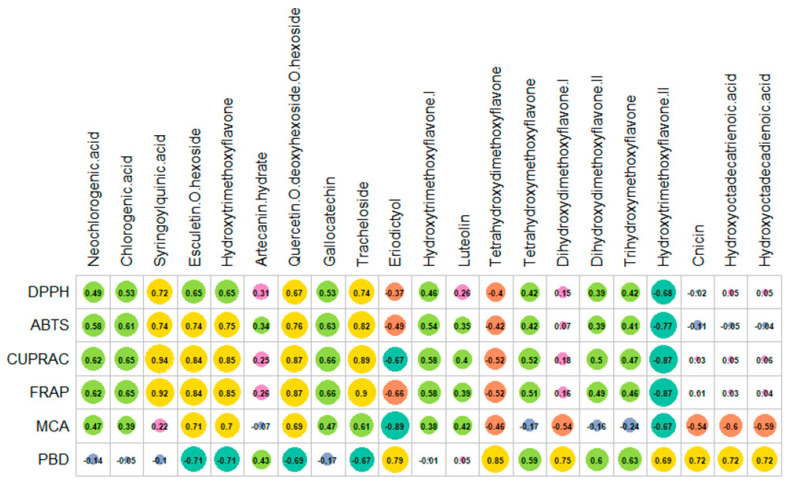
Pearson correlation between the antioxidant activity and individual phytochemicals.

To determine the connection, a Pearson correlation of the chemical profiles and biological abilities was undertaken. As depicted in Figure 4, it was observed that the FRAP, CUPRAC, and ABTS activities varied positively depending on the concentration of syringoylquinic acid, tracheloside, esculetin-*O*-hexoside, quercetin-*O*-deoxyhexoside-*O*-hexoside, and hydroxytrimethoxyflavone. Syringoylquinic acid, quercetin-*O*-deoxyhexoside-*O*-hexoside, and tracheloside appeared to be involved in DPPH scavenging activity. In addition, gallocatechin played a potent role in ABTS scavenging activity. Consistent with our findings, these compounds were labeled as natural antioxidants in previous studies [35,37,38,39,40]. Additionally, a significant correlation between the total bioactive constituents and antioxidant effects, particularly free radical scavenging and reducing power, has been reported in several studies on members of the *Artemisia* genus [41,42,43].

The phosphomolybdenum (PBD) method is also considered as a reducing power assay based on the reduction of Mo(VI) to Mo(V) by antioxidants in an acidic environment. However, this test assesses the total antioxidant capacity as not only phenolic, but also non-phenolic (tocopherol, ascorbic acid) antioxidants can act as reducing agents. Contrary to radical scavenging and reducing power assays, in the PBD method, the best activity was detected for the dichloromethane extract, with 2.11 mmol TE/g. In addition, the other three extracts (hexane, methanol, and hydroalcoholic) showed similar efficacy (*p* > 0.05). In the correlation analysis, the presence of some compounds (eriodictyol, tetrahydroxydimethoxyflavone, dihydroxydimethoxyflavone I, hydroxytrimethoxyflavone II, cnicin, and both fatty acid compounds (hydroxyoctadecatrienoic acid, hydroxyoctadecadienoic acid) could be assigned to the observed PBD activity. Finally, in our study, the chelation of transition metals, which relates to the hampering of hydroxyl radical production, was measured. As seen in Table 3, the strongest metal chelating abilities were provided by the hydroalcoholic (22.25 mg EDTAE/g) and water (21.61 mg EDTAE/g) extracts. Other extracts, namely hexane, dichloromethane, and methanol had similar metal chelating effects (*p* > 0.05). In the correlation analysis, several compounds such as quercetin-*O*-deoxyhexoside-*O*-hexoside, esculetin-*O*-hexoside and hydroxytrimethoxyflavone were related to the observed metal chelating activities. These compounds have also been described in the literature as metal chelating agents [44,45,46,47,48]. Taken together, *A. campestris* subsp. *lednicensis* might be considered as a source of health-promoting compounds with potential use in the development of functional ingredients.

### 2.4. Enzyme Inhibitory Properties

**Figure 5:**
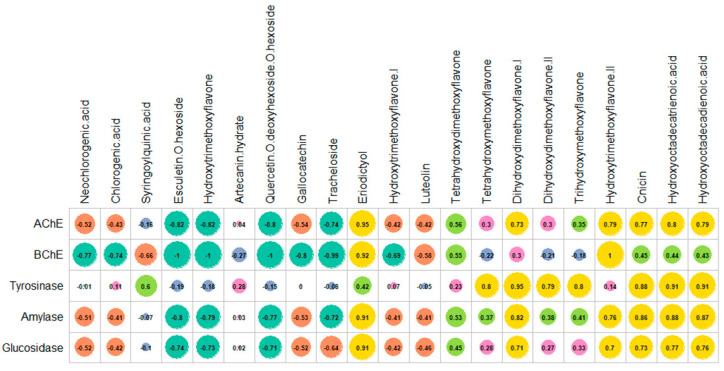
Pearson correlation between enzyme inhibitory activity and individual phytochemicals.

In the last decade, the term enzyme inhibition has gained scientific interest. With the increase in the human population, people need effective therapeutic strategies against serious human ailments called “global health diseases”. For example, the prevalence of Alzheimer’s disease increased from 20.2 million in 1990 to 43.8 million in 2006 [49]; a similar trend was also observed in the case of diabetes mellitus [50]. Considering this information, enzymes are believed to be the cornerstone in alleviating such diseases. Several key enzymes that are frequently screened in the enzyme inhibition assays include cholinesterase for Alzheimer’s, amylase for diabetes, and lipase for obesity. In this respect, several inhibitors have been chemically designed, but many of them exhibit various side effects [51,52]. Thus, safe and effective inhibitors derived from natural sources represent a promising research direction. In the current work, the enzyme inhibitory abilities of *A. campestris* subsp. *lednicensis* extracts against cholinesterase, tyrosinase, amylase, and glucosidase were assessed. The obtained results are presented in Table 4. The strongest AChE inhibitory ability was observed for dichloromethane extract (3.64 mg GALAE/g), followed by hexane (3.26 mg GALAE/g), methanol (2.66 mg GALAE/g), hydroalcoholic (1.21 mg GALAE/g), and water extracts (0.15 mg GALAE/g). Regarding the BChE inhibitory effect, two extracts (hexane and dichloromethane) were active against the enzyme, while the polar extracts showed no inhibitory activity. In the correlation analysis, eriodictyol and hydroxytrimethoxyflavone II strongly correlated with the observed anti-cholinesterase inhibitory activity (Figure 5). Our results also agree with the data reported by Uddin et al. [53] and Uriarte-Pueyo and Calvo [54]. Tyrosinase plays a key role in the synthesis of melanin and is therefore a checkpoint in the treatment of hyperpigmentation [55]. The highest tyrosinase inhibitory activity was displayed by the methanol extract (48.60 mg KAE/g), while the weakest was shown for the water extract (18.62 mg KAE/g) (Table 4). As depicted in Figure 4, flavone derivatives were strongly correlated with the demonstrated tyrosinase inhibitory effects. These data are also supported by findings from several studies that reported significant tyrosinase inhibitory abilities of flavonoids and their derivatives [56,57]. In both the amylase and glucosidase inhibition assays, the dichloromethane extract was the most active, followed by the hexane, methanol, hydroalcoholic, and water extracts (Table 4). The observed anti-amylase and anti-glucosidase abilities could be attributed to the presence of several compounds, among which special attention must be paid to eriodictyol. In fact, literature data support the potential of eriodictyol in the treatment of diabetes due to its insulin secretagogue properties [58].

### 2.5. Multivariate Analysis

**Figure 6:**
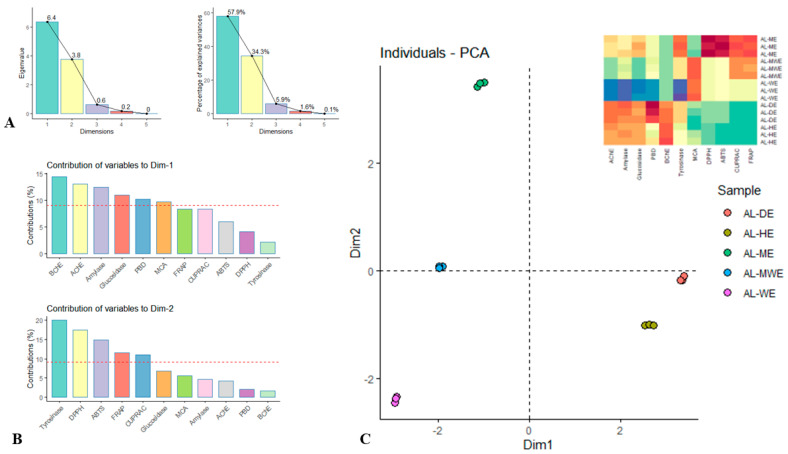
Principal component analysis on the biological activities of the *A. campestris* subsp. *lednicensis* aerial part extracts. (**A**) Eigenvalue and percentages of the explained variances of each dimension. (**B**) Contribution of variables on the dimensions of PCA. (**C**) Scatter plot showing the distribution of the samples in the two retained dimensions.

PCA is a common statistical tool to analyze compositional data in biopharmaceutical studies [59]. In this study, PCA was used to test the similarity of extraction solvents in terms of their antioxidant and enzyme inhibition activity. First, the number of significant dimensions was obtained based on the Kaiser criterion [60]. Accordingly, PCA showed that about 92% of the data variation could be explained by a total of two dimensions with proportions of 57.9% (Dim1) and 34.3% (Dim2) (Figure 6A). The remaining dimensions made very small contributions and were not retained for the following analysis. From the data in Figure 6B, it can be shown that dimension 1 (Dim1) predominantly discriminated the samples for both neurodegenerative and diabetes-related enzymes. In contrast, dimension 2 (Dim2) differentiated the samples according to their potential to inhibit the tyrosinase enzyme and scavenge DPPH and ABTS radicals. After describing the dimensions, a scatterplot of Dim1 vs. Dim2 was examined (Figure 6C). The extracts obtained with non-polar solvents (dichloromethane and hexane) were separated from the polar solvents (methanol, hydroalcoholic, water) along the first dimension. The dichloromethane and hexane extracts were very close and showed remarkable enzyme inhibitory activity. In contrast, the three polar solvent extracts were clearly separated from each other. Methanol extracts had excellent antioxidant activity, followed by the hydroalcoholic and water extracts.

### 2.6. Inhibitory Activity on the Cytokine Secretion

**Figure 7:**
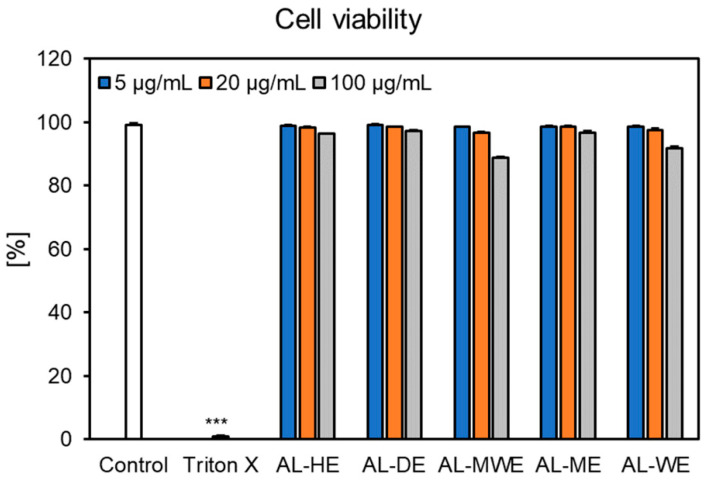
Neutrophil viability (%) after treatment with *Artemisia campestris* subsp. *lednicensis* extracts at concentrations of 5–100 μg/mL and Triton X-100 (0.1%). Results are presented as the mean ± SEM of three separate experiments performed with cells isolated from six independent donors; *** *p* < 0.001 vs. non-stimulated control. Legend: AL-HE, *A. campestris* subsp. *lednicensis* hexane extract; AL-DE, *A. campestris* subsp. *lednicensis* dichloromethane extract; AL-ME, *A. campestris* subsp. *lednicensis* methanol extract; AL-MWE, *A. campestris* subsp. *lednicensis* 50% methanol extract; AL-WE, *A. campestris* subsp. *lednicensis* water extract.

Neutrophils play a significant role in the initiation, progression, and resolution of the inflammatory response in the human body. Biofunctional ingredients endowed with both antioxidant and anti-inflammatory properties were prompted to reduce the risks of modern lifestyle diseases [61,62]. Considering that the *A. campestris* subsp. *lednicensis* extracts acted as significant antioxidant and anti-enzymatic effectors, we aimed to assess their influence on the inflammatory response. Thus, the neutrophils derived from healthy volunteers were stimulated with LPS, followed by the measurement of key pro-inflammatory cytokine (IL-1*β*, TNF-*α* and IL-8) levels in the presence of *Artemisia* extracts. Preliminary studies showed that over the tested concentration range (5–100 μg/mL), most samples exhibited no cytotoxicity toward human neutrophils (cell viabilities >96.38%), except for the hydroalcoholic and water extracts, which slightly altered their viability (88.74% and 91.87%, respectively, at 100 μg/mL) but still not significantly when compared to the non-stimulated control (Figure 7).

Neutrophils showed good viability and LPS stimulation promoted the secretion of functionally pro-inflammatory cytokines (e.g., TNF-α, IL-1*β,* and IL-8) (Figure 6). Interleukin-1*β* is the main pyrogenic molecule produced by leucocytes in response to noxious stimuli and promotes the secretion of adhesion molecules and thrombogenic mediators as well as the production of pro-inflammatory and tissue-remodeling enzymes [63]. After its release by neutrophils and macrophages, IL-8 acts as a chemotactic agent for basophils and lymphocytes and promotes their degranulation, followed by endothelial adhesion [61]. Synthetized in neutrophils, macrophages, natural killers, and mast cells, TNF-*α* induces apoptosis and modulates the expression of stress-activated protein kinases and nuclear factor kappa-light-chain-enhancer of activated B cells (NF-*κ*B) [64]. Thus, the suppression of IL-1*β*, IL-8, and TNF-α could be targeted in both acute and chronic inflammatory processes.

**Figure 8:**
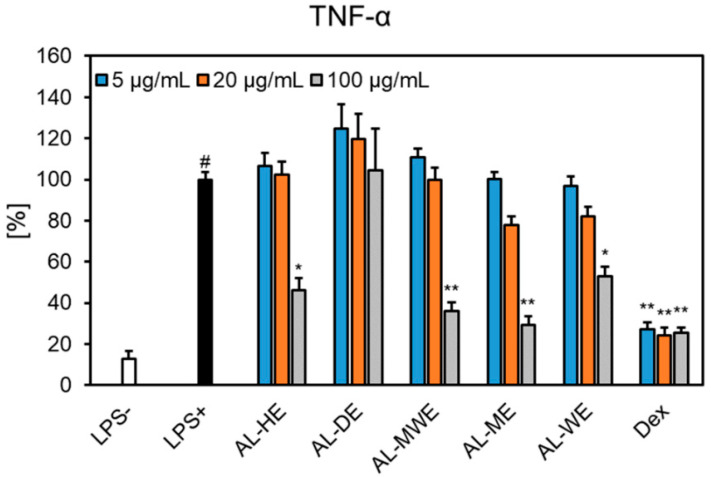
The effects of *Artemisia campestris* subsp. *lednicensis* extracts (5–100 μg/mL) and dexamethasone (Dex) (0.01–1 µM) on TNF-*α* release in LPS-stimulated (100 ng/mL) neutrophils. Results are presented as the mean ± SEM of three separate experiments performed with cells isolated from six independent donors; # *p* < 0.001 vs. non-stimulated control (LPS-); * *p* < 0.05; ** *p* < 0.001 vs. stimulated control (LPS+). Abbreviations: AL-HE, *A. campestris* subsp. *lednicensis* hexane extract; AL-DE, *A. campestris* subsp. *lednicensis* dichloromethane extract; AL-ME, *A. campestris* subsp. *lednicensis* methanol extract; AL-MWE, *A. campestris* subsp. *lednicensis* 50% methanol extract; AL-WE, *A. campestris* subsp. *lednicensis* water extract; LPS, lipopolysaccharide.

**Figure 9:**
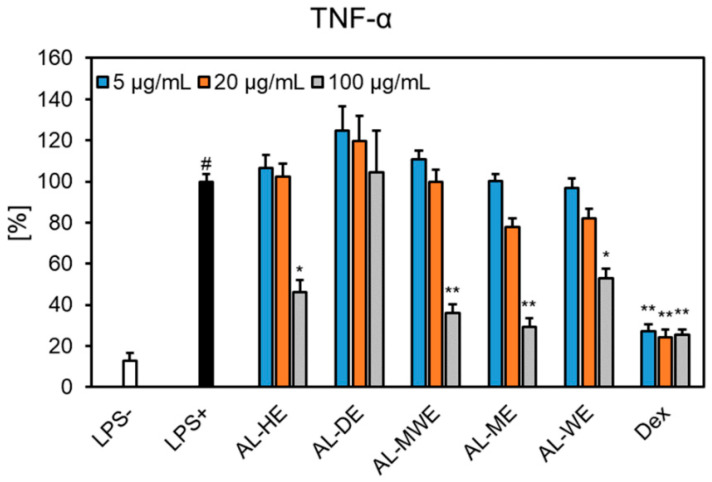
The effects of *Artemisia campestris* subsp. *lednicensis* extracts (5–100 μg/mL) and dexamethasone (Dex) (0.01–1 µM) on IL-1*β* release in LPS-stimulated (100 ng/mL) neutrophils. Results are presented as the mean ± SEM of three separate experiments performed with cells isolated from six independent donors; # *p* < 0.001 vs. non-stimulated control (LPS-); * *p* < 0.05; ** *p* < 0.001 vs. stimulated control (LPS+). Abbreviations: AL-HE, *A. campestris* subsp. *lednicensis* hexane extract; AL-DE, *A. campestris* subsp. *lednicensis* dichloromethane extract; AL-ME, *A. campestris* subsp. *lednicensis* methanol extract; AL-MWE, *A. campestris* subsp. *lednicensis* 50% methanol extract; AL-WE, *A. campestris* subsp. *lednicensis* water extracts; LPS, lipopolysaccharide.

Our study showed that *A. campestris* subsp. *lednicensis* extracts inhibited, with different degrees of potency, the release of cytokines in LPS-stimulated neutrophils. Except for the dichloromethane extract, all samples significantly inhibited the secretion of TNF-*α* (Figure 8). At 100 μg/mL, the following decreasing order of inhibition was observed: methanol extract (29.05% of LPS+ control) > hydroalcoholic extract (35.86% of LPS+ control) > hexane extract (45.98% of LPS+ control) > water extract (53.08% of LPS+ control). One must note that the effects displayed by the methanol extract were comparable to those of the positive control dexamethasone at 1 μM (25.30% of LPS + control). The investigated samples did not interfere with the production of IL-1*β* and IL-8 (Figure 8 and Figure 9). Our results are in agreement with previous in vitro and in vivo studies that reported on the ability of *Artemisia* species to impair the release of pro-inflammatory cytokines (e.g., TNF-*α,* IL-1, IL-6, IL-8, IL-11) [14,65]. Moreover, sesquiterpene lactones are acknowledged to act as anti-inflammatory and immunomodulatory effectors and their underlying mechanism relates to the inhibition of the NF-*κ*B pathway [66].

**Figure 10:**
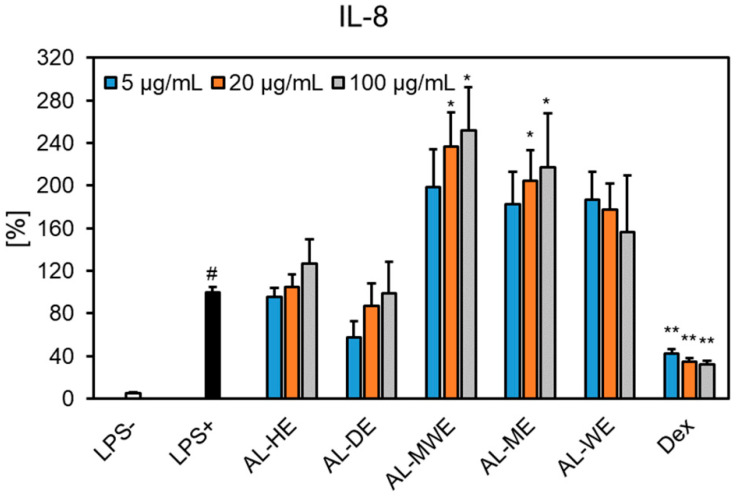
The effects of *Artemisia campestris* subsp. *lednicensis* extracts (5–100 μg/mL) and dexamethasone (Dex) (0.01–1 µM) on IL-8 production in LPS-stimulated (100 ng/mL) neutrophils. Results are presented as the mean ± SEM of three separate experiments performed with cells isolated from six independent donors; # *p* < 0.001 vs. non-stimulated control (LPS-); * *p* < 0.05; ** *p* < 0.001 vs. stimulated control (LPS+). Abbreviations: AL-HE, *A. campestris* subsp. *lednicensis* hexane extract; AL-DE, *A. campestris* subsp. *lednicensis* dichloromethane extract; AL-ME, *A. campestris* subsp. *lednicensis* methanol extract; AL-MWE, *A. campestris* subsp. *lednicensis* 50% methanol extract; AL-WE, *A. campestris* subsp. *lednicensis* water extract; LPS, lipopolysaccharide.

As depicted in Figure 9 and Figure 10, the investigated *Artemisia* extracts tended to induce the overproduction of IL-1*β* and IL-8 in the LPS-stimulated neutrophils. Therefore, *A. campestris* subsp. *lednicensis* extracts might not protect neutrophils from the deleterious effects of these mediators, which are involved in the initiation and progression of the inflammation process. Moreover, the overproduction of such pro-inflammatory cytokines or the “inflammatory cascades” is the first response of host defense in the case of invading pathogens [67]. Thereby, phytoconstituents from the investigated *Artemisia* extracts could act as anti-infectious agents and consequently, an assessment of their antimicrobial was further undertaken.

### 2.7. Antimicrobial Properties

The results of the anti-inflammatory screening prompted us to explore the putative antimicrobial properties of *A. campestris* subsp. *lednicensis* extracts. The criteria proposed by de Oliveira Lima et al. [68] were used to rank the antimicrobial screening results: strong activity (MIC 50–500 mg/L), moderate activity (500 mg/L > MIC < 1500 mg/L), and weak activity (MIC > 1500 mg/L). Overall, the investigated *Artemisia* extracts showed weak activity toward the tested strains (Table 5). *H. pylori* was the most sensitive, with the hexane, hydroalcoholic and water extracts displaying a moderate activity toward this Gram-negative bacterium (MIC = 625 mg/L). The anti-*H. pylori* activity of *Artemisia* species was previously reported. The water extract of *A. douglasiana* and its main constituent dehydroleucodine, a guaianolide-type sesquiterpene lactone, displayed significant inhibitory effects against the reference and clinical isolates of *H. pylori*, with MIC values of 1–8 mg/L and 60–120 mg/L, respectively [69]. In addition, an aqueous extract of *A. ludoviciana* subsp. *mexicana* was shown to exert anti-*H. pylori* properties (MIC = 250 mg/L). Further fractionation of the extract afforded the isolation of the sesquiterpene lactone estafiatin and polymethoxylated flavone eupatilin, which were the main bioactive constituents responsible for the inhibition of *H. pylori* (MIC values of 15.6 and 31.2 mg/L, respectively) [70]. In the case of *A. campestris* subsp. *lednicensis*, the LC-MS/MS analysis annotated the presence of both sesquiterpenes and polyhydroxy- and poly-methoxylated flavone derivatives in the extracts displaying anti-*H. pylori* activity. Therefore, we can hypothesize that these constituents might contribute to the observed effects, but the synergistic effects among various classes of compounds identified in the studied extracts (e.g., phenolic acids, flavonoids, sesquiterpenes, coumarins, lignans and fatty acids) cannot be excluded.

## 3. Materials and Methods

### 3.1. Chemicals

1,1-Diphenyl-2-picrylhydrazyl (DPPH), 2′-azino-bis(3-ethylbenzothiazoline-6-sulfonic acid (ABTS), 2,4,6-tris(2-pyridyl)-s-triazine (TPTZ), 5,5-dithio-bis(2-nitrobenzoic) acid (DTNB), α-glucosidase (EC. 3.2.1.20, from *Saccharomyces cerevisiae*), *α*-amylase (EC. 3.2.1.1, from porcine pancreas), acarbose, acetylthiocholine iodide (ATChI), ammonium acetate, ammonium molybdate, butyrylthiocholine chloride (BTChI), cupric chloride, dichloromethane, electric eel acetylcholinesterase (AChE) (type-VI-S, EC 3.1.1.7), ethylenediaminetetraacetate (EDTA), ferric chloride, ferrozine, ferrous sulfate hexahydrate, Folin–Ciocalteu reagent, galantamine, gallic acid, hexane, horse serum butyrylcholinesterase (BChE) (EC 3.1.1.8), hydrochloric acid, kojic acid, lipopolysaccharide (LPS, from *Escherichia coli* 0111:B4), methanol, neocuproine, rutin, sodium hydroxide, sodium molybdate, sodium nitrate, sodium carbonate, trolox, and tyrosinase (EC1.14.18.1, mushroom) were obtained from Merck KGaA (Darmstadt, Germany). Citric acid, glucose for citrate dextrose solution (ACD), and sodium citrate tribasic dihydrate were purchased from Chempur (Piekary Śląskie, Poland). Chloramphenicol, cycloheximide, glucose, RPMI 1640 medium, and terbinafine hydrochloride were acquired from Sigma-Aldrich (Steinheim, Germany). Dextran from *Leuconostoc mesenteroides*, propidium iodide (PI), dexamethasone (Dex), Triton X-100, and amphotericin B were bought from Sigma-Aldrich (Saint Louis, MO, USA). Fetal bovine serum (FBS), RPMI 1640 enriched by 10 mM HEPES (4-(2-hydroxyethyl)-1-piperazineethanesulfonic acid), and 2 mM L-glutamine as well as penicillin-streptomycin were obtained from Biowest (Nauillé, France). Pancoll Human (P04-601000) was purchased from PAN-Biotech (Aidenbach, Germany). Liquid chromatography (LC) grade acetonitrile, formic acid, and water were obtained from Merck KGaA (Darmstadt, Germany). Phosphate buffered saline (PBS) was purchased from Gibco (Gibco, HK, China), whereas the calcium-free PBS was from Biomed (Lublin, Poland). Buffy coats, used for neutrophil isolation, were provided by the Warsaw Blood Donation Centre (Warsaw, Poland). Human ELISA sets (TNF-*α,* IL-1*β*, IL-8) were bought from BD Biosciences (Erembodegem, Belgium).

### 3.2. Plant Material and Extraction

The aerial parts of *Artemisia campestris* subsp. *lednicensis* were collected during September 2020 from Aroneanu Lake area, Iasi county, Romania (GPS coordinates 47.203117, 27.593671). The plant material was authenticated by Dr. Constantin Mardari and Dr. Adrian Oprea, Botanic Garden “Anastasie Fatu”, Iasi, Romania. A voucher specimen (ACL/2021) was deposited in the Department of Pharmacognosy, “Grigore T. Popa” University of Medicine and Pharmacy Iasi (Romania). The aerial parts were dried, ground, and then 5 g were separately extracted with solvents of different polarities (hexane, dichloromethane, methanol, 50% methanol, and water) by ultrasonication (three cycles of 30 min. each, at room temperature). The extracts were evaporated to dryness under vacuum (the obtained yields are shown in Table 1) and kept at −20 °C until subsequent experiments.

### 3.3. Phytochemical Analysis

The total phenolic and flavonoid content were evaluated using the Folin–Ciocalteu and AlCl_3_ tests, respectively [71]. Results were expressed as gallic acid equivalents (mg GAE/g dry extract) and rutin equivalents (mg RE/g dry extract) for these assays. The LC-HRMS/MS analysis was performed following the methodology extensively described in Trifan et al. [18].

### 3.4. Antioxidant Assays

Antioxidant assays were performed using previously described methods [72,73]. The antioxidant potential was reported as follows: mg Trolox equivalents (TE)/g extract in the DPPH and ABTS radicals scavenging, CUPRAC, FRAP, and MCA tests; mmol TE/g extract in the PBD assay.

### 3.5. Enzyme Inhibitory Assays

The enzyme inhibition experiments were performed following the previously described methodologies [72,73]. Amylase and glucosidase inhibition were expressed as mmol acarbose equivalents (ACAE)/g extract, while AChE and BChE inhibition was expressed as mg galanthamine equivalents (GALAE)/g extract. Tyrosinase inhibition was expressed as mg kojic acid equivalents (KAE)/g extract.

### 3.6. Cytokine Secretion in Human Neutrophils

The influence of the tested samples on cytokine secretion was assessed in human neutrophils using a methodology previously described [61]. Briefly, neutrophils were isolated by dextran sedimentation and centrifugation in a Pancoll gradient, providing the neutrophil preparation (purity >97%). The *A. campestris* subsp. *lednicensis* extracts (5, 20, and 100 μg/mL) were added 30 min before stimulation with LPS (100 ng/mL). Neutrophils (2 × 10^6^/mL) were co-incubated with extracts for 18 h in RPMI 1640 medium supplemented with 10% FBS and 1% penicillin-streptomycin. Then, the neutrophils were centrifuged and washed twice with calcium-free PBS, re-suspended in PI solution (0.5 μg/mL), and followed by incubation (15 min in the dark, at room temperature). Within one hour, the neutrophils were analyzed by flow cytometry (BD FACSVerse, BD Biosciences, San Jose, CA, USA). The viability of neutrophils was calculated as % = 100% − %PI+ cells; Triton X (0.1%) was the positive control. The release of cytokines (IL-1*β*, IL-8, and TNF-*α*) was evaluated by ELISA using a Synergy 4 microplate reader (BioTek, Winooski, VT, USA), following the manufacturer’s indications. The secretion of cytokines was expressed as a percentage of the cytokines released into the supernatant compared to the LPS+ stimulated cells; dexamethasone (0.01, 0.1, and 1 μM) was used as the positive control.

### 3.7. Antimicrobial Assays

The antimicrobial assays were performed by the microdilution method according to the European Committee on Antimicrobial Susceptibility Testing [74,75]. Mueller-Hinton broth and MH broth with 7% lysed horse blood were used for the growth of non-fastidious bacteria and *H. pylori*, respectively, whereas MH broth with 2% glucose was used for the growth of yeasts. RPMI 1640 medium 2% glucose buffered with 0.165 M MOPS and supplemented with cycloheximide 300 mg/L and chloramphenicol 50 mg/L was utilized for the growth of dermatophytes [74,75]. Minimum inhibitory concentrations (MIC) of the samples were determined in Gram-positive bacteria (*Staphylococcus aureus* ATCC 25923, *S. epidermidis* ATCC 12228, *Micrococcus luteus* ATCC 10240, *Enterococcus faecalis* ATCC 29212, *Bacillus subtilis* ATCC 6633, and *B. cereus* ATCC 10876); Gram-negative bacteria (*Salmonella* Typhimurium ATCC 14028, *Escherichia coli* ATCC 25922, *Proteus mirabilis* ATCC 12453, *Klebsiella pneumoniae* ATCC 13883, *Pseudomonas aeruginosa* ATCC 90271, and *Helicobacter pylori* ATCC 43504); yeasts (*Candida albicans* ATCC 2091, *C. glabrata* ATCC 90030, and *C. parapsilosis* ATCC 22019) and dermatophytes (*Trichophyton rubrum* ATCC 28188 and *T. mentagrophytes* ATCC 9533). All experiments were performed in triplicate.

### 3.8. Statistical Analysis

Antioxidant and enzyme inhibitory experiments were performed in triplicate, with the data presented as the mean ± standard deviation. The phytochemical dataset was logarithm transformed, scaled, and submitted to clustered image maps (CIMs). For CIMs, “Ward’s rule” and “Euclidean distance” were used. Then, the antioxidant and anti-enzymatic activities dataset was also scaled, centered, and submitted to the principal component analysis (PCA). Then, the relationship between phytochemical and antioxidant/ anti-enzymatic activities was evaluated by calculating the Pearson correlation coefficient. A Pearson’s coefficient greater than 0.7 was considered significant. CIMs, PCA, and correlation analysis were conducted under R v 4.1.2 software. The evaluation of neutrophil viability and cytokine release was determined in three independent experiments performed with cells isolated from six separate donors. Data were expressed as the mean ± standard error of the mean. Statistical analysis was evaluated by one-way ANOVA, with Tukey’s and Dunnett’s multiple comparison tests. If necessary, non-parametric methods such as the Mann–Whitney test were employed; *p* < 0.05 was considered significant. Statistica 13 software (TIBCO Software Inc., Palo Alto, CA, USA) was employed.

## 4. Conclusions

We report herein for the first time a comprehensive metabolite and biological profiling of *Artemisia campestris* subsp. *lednicensis* aerial part extracts. By using a LC-HRMS/MS-based platform, 21 specialized metabolites belonging mostly to polyphenolic compounds were annotated such as twelve flavonoids (mostly polyhydroxy-/poly-methoxylated flavones), three phenolic acids (quinic acid derivatives), two sesquiterpene lactones, two fatty acids, one coumarin, and one lignan. PCA revealed that polar extracts (in particular the methanol extract) were more active than the non-polar extracts in the antioxidant assays (radical scavenging, metal reducing, chelating, and phosphomolybdenum). In contrast, the non-polar extracts exhibited higher anti-enzymatic activity compared to the polar extracts against key enzymes targeted in the management of chronic diseases such as Alzheimer’s (acetylcholinesterase, butyrylcholinesterase), type 2 diabetes mellitus (amylase, glucosidase), and skin disorders (tyrosinase). Furthermore, the *Artemisia* extracts were shown to downregulate the release of TNF-*α* in the LPS-stimulated human neutrophil model. Finally, moderate anti-*H. pylori* effects were displayed by the investigated samples.

Our study reveals *A. campestris* subsp. *lednicensis* as a valuable source of compounds endowed with important biological activities and brings additional data that support the pleiotropic pharmacology of the *Artemisia* genus. In conclusion, the obtained results represent a starting point in the further development of *A. campestris* subsp. *lednicensis* extracts as nutraceutical, cosmeceutical, and pharmaceutical ingredients addressing modern life-related diseases.

## Figures and Tables

**Table 1:** Extraction yields, total phenolic, and flavonoid content of *Artemisia campestris* subsp. *lednicensis* extracts.

Sample	Yield (%)	Total Phenolic Content (mg GAE/g)	Total Flavonoid Content (mg RE/g)
**AL-HE**	4.33	17.11 ± 0.27 ^e^	9.57 ± 0.14 ^c^
**AL-DE**	6.20	20.67 ± 0.52 ^d^	16.43 ± 0.37 ^b^
**AL-ME**	12.60	84.42 ± 0.33 ^b^	23.13 ± 0.73 ^a^
**AL-MWE**	22.59	104.00 ± 0.69 ^a^	15.08 ± 0.26 ^b^
**AL-WE**	23.55	71.73 ± 0.78 ^c^	8.92 ± 0.31 ^c^

Results are given as the mean ± standard deviation of three replicates; significant differences in the investigated samples (*p* < 0.05) are indicated by different letters within columns. AL-HE, *A. campestris* subsp. *lednicensis* hexane extract; AL-DE, *A. campestris* subsp. *lednicensis* dichloromethane extract; AL-ME, *A. campestris* subsp. *lednicensis* methanol extract; AL-MWE, *A. campestris* subsp. *lednicensis* 50% methanol extract; AL-WE, *A. campestris* subsp. *lednicensis* water extract; GAE, gallic acid equivalents; RE, rutin equivalents.

**Table 2:** LC-HRMS/MS-based metabolite profiling of the *A. campestris* subsp. *lednicensis* aerial part extracts.

No.	Proposed Identity	Class	TR (min)	[M-H]^−^ (*m/z*)	MF	MS/MS (*m/z*)	AL-HE	AL-DE	AL-ME	AL-MWE	AL-WE
**1**	Neochlorogenic acid *	Phenolic acid	9.8	353.0893	C_16_H_18_O_9_	191.0484, 179.0252, 135.0370	–	×	×	×	×
**2**	Chlorogenic acid *	Phenolic acid	13.2	353.0893	C_16_H_18_O_9_	191.0568, 173.0429, 135.0461	–	×	×	×	×
**3**	Syringoylquinic acid	Phenolic acid	14.6	371.0969	C_16_H_20_O_10_	353.0882, 339.0740, 209.0675, 179.0358	–	–	×	×	–
**4**	Esculetin-*O*-hexoside	Coumarin	14.9	339.0715	C_15_H_16_O_9_	177.0233, 149.0157, 133.0217, 105.0327	–	–	×	×	×
**5**	Hydroxytrimethoxyflavone (e.g., salvigenin)	Flavonoid	16.4	327.0847	C_18_H_16_O_6_	241.0099, 177.0415, 151.0075	–	–	×	×	×
**6**	Artecanin hydrate	Sesquiterpene	19.6	295.1169	C_15_H_20_O_6_	251.1300, 207.1409, 189.1280, 151.0831	×	×	×	×	×
**7**	Quercetin-*O*-deoxyhexoside-*O*-hexoside (e.g., rutin)	Flavonoid	23.1	609.1476	C_27_H_30_O_16_	300.0287, 271.0255, 151.0035	–	–	×	×	×
**8**	Gallocatechin	Flavonoid	25.0	305.0671	C_15_H_14_O_7_	225.1144, 147.0823	–	×	×	×	×
**9**	Tracheloside	Lignan	26.9	549.1985	C_27_H_34_O_12_	387.1681, 207.1037, 179.0375, 161.0251	–	–	×	×	×
**10**	Eriodictyol	Flavonoid	29.0	287.0567	C_15_H_12_O_6_	151.0046, 135.0479	×	×	×	×	×
**11**	Dihydroxytrimethoxyflavone I (e.g., penduletin)	Flavonoid	30.1	343.0813	C_18_H_16_O_7_	328.0382, 313.0382, 298.0133, 285.0421, 270.0199, 255.0318, 242.0284	–	×	×	×	×
**12**	Luteolin *	Flavonoid	31.0	285.0400	C_15_H_10_O_6_	175.0386, 133.0313	–	×	×	×	×
**13**	Tetrahydroxydimethoxyflavone (e.g., eupatolitin)	Flavonoid	31.5	345.0602	C_17_H_14_O_8_	330.0402, 315.0188, 287.0296, 259.0301, 259.0301, 215.0351, 175.0091, 149.0308, 121.0326	–	×	–	–	–
**14**	Tetrahydroxymethoxyflavone (e.g., rhamnetin)	Flavonoid	31.7	315.0509	C_16_H_12_O_7_	300.0327, 271.0269, 255.0312, 243.0322, 227.0356, 215.0350, 171.0409, 147.0202	–	×	×	×	–
**15**	Dihydroxydimethoxyflavone I (e.g., rhamnazin)	Flavonoid	33.9	329.0678	C_17_H_14_O_7_	314.0456, 299.0241, 271.0279, 271.0272, 243.0312, 227.0430, 215.0360, 199.0421, 185.0236, 161.0264, 151.0068, 133.0347	×	×	×	×	–
**16**	Dihydroxydimethoxyflavone II (e.g., eupalitin)	Flavonoid	35.4	329.0678	C_17_H_14_O_7_	314.0456, 299.0241, 271.0279, 271.0272, 243.0312, 227.0430, 215.0360, 199.0421, 185.0236, 161.0264, 151.0068, 133.0347	–	×	×	–	–
**17**	Trihydroxymethoxyflavone (e.g., diosmetin)	Flavonoid	37.0	299.0566	C_16_H_12_O_6_	284.0259, 255.0179, 239.0292, 227.0330, 151.0077, 133.0252	–	×	×	×	–
**18**	Dihydroxytrimethoxyflavone II (e.g., eupatilin)	Flavonoid	37.6	343.0813	C_18_H_16_O_7_	328.0382, 313.0382, 298.0133, 285.0421, 270.0199, 255.0318, 242.0284	×	×	–	–	–
**19**	Cnicin	Sesquiterpene	39.4	377.1617	C_20_H_26_O_7_	295.1213, 251.1322, 189.1257, 151.07060	×	×	×	×	–
**20**	Hydroxyoctadecatrienoic acid	Fatty acid	46.8	293.2118	C_18_H_30_O_3_	275.1973, 224.1359, 195.1381	×	×	×	×	–
**21**	Hydroxyoctadecadienoic acid	Fatty acid	49.3	295.2269	C_18_H_32_O_3_	277.2162, 195.1407, 171.1029	×	×	×	×	–

AL-HE, *A. campestris* subsp. *lednicensis* hexane extract; AL-DE, *A. campestris* subsp. *lednicensis* dichloromethane extract; AL-ME, *A. campestris* subsp. *lednicensis* methanol extract; AL-MWE, *A. campestris* subsp. *lednicensis* 50% methanol extract; AL-WE, *A. campestris* subsp. *lednicensis* water extract; * confirmed by standard. The LC-HRMS/MS analyses were performed on a Phenomenex Gemini C18 column (2 mm × 100 mm, 3 μm); mobile phase 0.1% formic acid in water (A) and 0.1% formic acid in acetonitrile (B); gradient 5–60% B (0–45 min), 95% B (46–55 min); flow rate 0.2 mL/min. The following MS conditions were applied: negative ionization mode; *m/z* range 100–1000; gas (N_2_) temperature 275 °C; N_2_ flow 10 L/min; nebulizer 35 psi; sheath gas temperature 325 °C; sheath gas flow rate 12 L/min; capillary voltage 4000 V; nozzle voltage 1000 V; skimmer 65 V; fragmentor 140 V; collision-induced dissociation energies 10 and 30 V.

**Table 3:** The antioxidant activity of *Artemisia campestris* subsp. *lednicensis* extracts.

Sample	DPPH (mg TE/g)	ABTS (mg TE/g)	CUPRAC (mg TE/g)	FRAP (mg TE/g)	Metal Chelating (mg EDTAE/g)	Phosphomolybdenum (mmol TE/g)
**AL-HE**	0.71 ± 0.07 ^d^	20.57 ± 1.06 ^d^	38.56 ± 0.62 ^d^	21.68 ± 0.51 ^d^	13.64 ± 1.52 ^b^	1.46 ± 0.13 ^b^
**AL-DE**	7.63 ± 0.49 ^c^	44.96 ± 1.95 ^c^	44.00 ± 0.94 ^d^	25.04 ± 0.77 ^d^	12.88 ± 0.99 ^b^	2.11 ± 0.11 ^a^
**AL-ME**	213.68 ± 7.30 ^a^	356.35 ± 9.46 ^a^	311.21 ± 3.66 ^a^	202.34 ± 3.26 ^a^	13.35 ± 0.33 ^b^	1.42 ± 0.02 ^b^
**AL-MWE**	61.74 ± 0.12 ^b^	152.40 ± 0.21 ^b^	275.79 ± 0.47 ^b^	166.59 ± 3.85 ^b^	22.25 ± 0.24 ^a^	1.41 ± 0.06 ^b^
**AL-WE**	58.78 ± 0.09 ^b^	151.76 ± 0.15 ^b^	143.32 ± 1.08 ^c^	91.93 ± 0.30 ^c^	21.61 ± 0.34 ^a^	0.92 ± 0.01 ^c^

Results are given as the mean ± standard deviation of three replicates; significant differences in the investigated samples (*p* < 0.05) are indicated by different letters within columns. AL-HE, *A. campestris* subsp. *lednicensis* hexane extract; AL-DE, *A. campestris* subsp. *lednicensis* dichloromethane extract; AL-ME, *A. campestris* subsp. *lednicensis* methanol extract; AL-MWE, *A. campestris* subsp. *lednicensis* 50% methanol extract; AL-WE, *A. campestris* subsp. *lednicensis* water extract; EDTAE, EDTA equivalents; TE, trolox equivalents.

**Table 4:** Enzyme inhibitory activity of *Artemisia campestris* subsp. *lednicensis* extracts.

Sample	AChE (mg GALAE/g)	BChE (mg GALAE/g)	Tyrosinase (mg KAE/g)	Amylase (mmol ACAE/g)	Glucosidase (mmol ACAE/g)
**AL-HE**	3.26 ± 0.06 ^b^	3.18 ± 0.06 ^a^	35.89 ± 2.11 ^c^	0.35 ± 0.01 ^a^	2.16 ± 0.03 ^a^
**AL-DE**	3.64 ± 0.09 ^a^	2.82 ± 0.15 ^b^	41.53 ± 0.34 ^b^	0.38 ± 0.02 ^a^	2.21 ± 0.02 ^a^
**AL-ME**	2.66 ± 0.08 ^c^	n.a.	48.60 ± 0.67 ^a^	0.29 ± 0.02 ^b^	2.06 ± 0.13 ^b^
**AL-MWE**	1.21 ± 0.02 ^d^	n.a.	40.38 ± 0.48 ^b^	0.20 ± 0.01 ^c^	0.88± 0.02 ^c^
**AL-WE**	0.15 ± 0.05 ^e^	n.a.	18.62 ± 1.28 ^d^	0.05 ± 0.00 ^d^	0.43 ± 0.03 ^d^

Results are given as the mean ± standard deviation of three replicates; significant differences in the investigated samples (*p* < 0.05) are indicated by different letters within columns. ACAE, acarbose equivalents; AL-HE, *A. campestris* subsp. *lednicensis* hexane extract; AL-DE, *A. campestris* subsp. *lednicensis* dichloromethane extract; AL-ME, *A. campestris* subsp. *lednicensis* methanol extract; AL-MWE, *A. campestris* subsp. *lednicensis* 50% methanol extract; AL-WE, *A. campestris* subsp. *lednicensis* water extract; GALAE, galanthamine equivalents; KAE, kojic acid equivalents; n.a., not active.

**Table 5:** Antimicrobial properties of the *Artemisia campestris* subsp. *lednicensis* extracts.

Microorganism	MIC (mg/L)					
	AL-HE	AL-DE	AL-ME	AL-MWE	AL-WE	Positive Control
**Gram-positive bacteria**						Vancomycin
*Staphylococcus aureus* ATCC 25923	>5000	5000	5000	>5000	5000	0.98
*Staphylococcus epidermidis* ATCC 12228	>5000	5000	5000	>5000	5000	0.98
*Micrococcus luteus* ATCC 10240	>5000	5000	5000	>5000	2500	0.12
*Enterococcus faecalis* ATCC 29212	>5000	>5000	>5000	>5000	>5000	1.95
*Bacillus subtilis* ATCC 6633	>5000	>5000	>5000	>5000	5000	0.24
*Bacillus cereus* ATCC 10876	>5000	5000	>5000	>5000	5000	0.98
**Gram-negative bacteria**						Ciprofloxacin
*Salmonella* Typhimurium ATCC 14028	>5000	>5000	>5000	>5000	>5000	0.061
*Escherichia coli* ATCC 25922	>5000	>5000	>5000	>5000	>5000	0.015
*Proteus mirabilis* ATCC 12453	>5000	5000	>5000	>5000	>5000	0.030
*Klebsiella pneumoniae* ATCC 13883	>5000	>5000	>5000	>5000	>5000	0.122
*Pseudomonas aeruginosa* ATCC 90271	>5000	>5000	>5000	>5000	>5000	0.488
*Helicobacter pylori* ATCC 43504	625	2500	5000	625	625	1.0 (Ofloxacin)
**Yeasts**						Nystatin
*Candida albicans* ATCC 2091	5000	5000	5000	2500	5000	0.48
*Candida parapsilosis* ATCC 22019	5000	5000	5000	2500	2500	0.24
*Candida glabrata* ATCC 90030	10,000	10,000	10,000	10,000	10,000	0.24
**Dermatophytes**						Terbinafine
*Trichophyton rubrum* ATCC 28188	>1000	>1000	>1000	>1000	>1000	0.031
*Trichophyton mentagrophytes* ATCC 9533	>1000	>1000	>1000	>1000	>1000	0.031

AL-HE, *A. campestris* subsp. *lednicensis* hexane extract; AL-DE, *A. campestris* subsp. *lednicensis* dichloromethane extract; AL-ME, *A. campestris* subsp. *lednicensis* methanol extract; AL-MWE, *A. campestris* subsp. *lednicensis* 50% methanol extract; AL-WE, *A. campestris* subsp. *lednicensis* water extract; MIC, minimum inhibitory concentration.

## Data Availability

Not applicable.

## References

[1] Abad M.J., Bedoya L.M., Apaza L., Bermejo P. (2012). The *Artemisia* L. genus: A review of bioactive essential oils. Molecules.

[2] Nigam M., Atanassova M., Mishra A.P., Pezzani R., Devkota H.P., Plygun S., Salehi B., Setzer W.N., Sharifi-Rad J. (2019). Bioactive compounds and health benefits of *Artemisia* species. Nat. Prod. Commun..

[3] Trendafilova A., Moujir L.M., Sousa P.M., Seca A.M. (2020). Research advances on health effects of edible *Artemisia* species and some sesquiterpene lactones constituents. Foods.

[4] Turi C.E., Shipley P.R., Murch S.J. (2014). North American *Artemisia* species from the subgenus Tridentatae (Sagebrush): A phytochemical, botanical and pharmacological review. Phytochemistry.

[5] Bora K.S., Sharma A. (2011). The genus *Artemisia*: A comprehensive review. Pharm. Biol..

[6] Feng X., Cao S., Qiu F., Zhang B. (2020). Traditional application and modern pharmacological research of *Artemisia annua* L.. Pharmacol. Ther..

[7] Bisht D., Kumar D., Kumar D., Dua K., Chellappan D.K. (2021). Phytochemistry and pharmacological activity of the genus *Artemisia*. Arch. Pharm. Res..

[8] Taleghani A., Emami S.A., Tayarani-Najaran Z. (2020). *Artemisia*: A promising plant for the treatment of cancer. Bioorg. Med. Chem..

[9] Pandey A.K., Singh P. (2017). The genus *Artemisia*: A 2012–2017 literature review on chemical composition, antimicrobial, insecticidal and antioxidant activities of essential oils. Medicines.

[10] Ali M., Abbasi B.H., Ahmad N., Khan H., Ali G.S. (2017). Strategies to enhance biologically active-secondary metabolites in cell cultures of *Artemisia*–current trends. Crit. Rev. Biotechnol..

[11] Ivanescu B., Lungu C., Vlase L., Gheldiu A.M., Grigorescu C., Corciova A. (2018). Bioactive compounds from *Artemisia campestris* L. subsp. campestris. Dementia.

[12] Dib I., El Alaoui-Faris F.E. (2019). *Artemisia campestris* L.: Review on taxonomical aspects, cytogeography, biological activities and bioactive compounds. Biomed. Pharmacother..

[13] Ghorab H., Laggoune S., Kabouche A., Semra Z., Kabouche Z. (2013). Essential oil composition and antibacterial activity of *Artemisia campestris* L. from Khenchela (Algeria). Der Pharm. Lett..

[14] Anibogwu R., Jesus K.D., Pradhan S., Pashikanti S., Mateen S., Sharma K. (2021). Extraction, isolation and characterization of bioactive compounds from *Artemisia* and their biological significance: A review. Molecules.

[15] The World Flora Online (WFO). http://www.worldfloraonline.org/taxon/wfo-0000097078.

[16] Ciocârlan V. (2000). Flora Ilustrată a României: Pteriodophyta et Spermatophyta.

[17] Clinciu R.R.A., Onofrei V., Robu T., Nastase S. (2015). Study opportunities for spreading and use of certain species of *Artemisia* present in Moldova. An. Univ. Din Oradea Fasc. Protecția Mediu..

[18] Trifan A., Zengin G., Sinan K.I., Sieniawska E., Sawicki R., Maciejewska-Turska M., Skalikca-Woźniak K., Luca S.V. (2022). Unveiling the Phytochemical Profile and Biological Potential of Five *Artemisia* Species. Antioxidants.

[19] Dhifallah A., Selmi H., Ouerghui A., Sammeri H., Aouini D., Rouissi H. (2022). Comparative Study of Phenolic Compounds and Antiradical Activities of Four Extracts of Tunisian *Artemisia herba alba*. Pharm. Chem. J..

[20] Tayyaba Batool Kazmi S., Naz I., Saniya Zahra S., Nasar H., Fatima H., Shuja Farooq A., Haq I.-u. (2022). Phytochemical analysis and comprehensive evaluation of pharmacological potential of *Artemisia brevifolia* Wall. ex DC. Saudi Pharm. J..

[21] Sun C., Wu Z., Wang Z., Zhang H. (2015). Effect of Ethanol/Water Solvents on Phenolic Profiles and Antioxidant Properties of Beijing Propolis Extracts. Evid. Based Complement. Alternat. Med..

[22] Sepahpour S., Selamat J., Abdul Manap M.Y., Khatib A., Abdull Razis A.F. (2018). Comparative Analysis of Chemical Composition, Antioxidant Activity and Quantitative Characterization of Some Phenolic Compounds in Selected Herbs and Spices in Different Solvent Extraction Systems. Molecules.

[23] Riahi L., Chograni H., Masmoudi A.S., Cherif A. (2021). Variability of phenolic compounds accumulation and bioactivity among Tunisian *Artemisia arborescens* L. genetic resources. Res. J. Biotechnol..

[24] Nataraj N., Hussain M., Ibrahim M., Hausmann A.E., Rao S.N.V., Kaur S., Khazir J., Mir B.A., Olsson S.B. (2022). Effect of Altitude on Volatile Organic and Phenolic Compounds of *Artemisia brevifolia* Wall ex Dc. From the Western Himalayas. Front. Ecol. Evol..

[25] Boukhalkhal S., Gourine N., Pinto D., Silva A.M.S., Yousfi M. (2020). UHPLC-DAD-ESI-MSn profiling variability of the phenolic constituents of *Artemisia campestris* L. populations growing in Algeria. Biocatal. Agric. Biotechnol..

[26] Jakovljevic M.R., Grujicic D., Vukajlovic J.T., Markovic A., Milutinovic M., Stankovic M., Vukovic N., Vukic M., Milosevic-Djordjevic O. (2020). In vitro study of genotoxic and cytotoxic activities of methanol extracts of *Artemisia vulgaris* L. and *Artemisia alba* Turra. S. Afr. J. Bot..

[27] Bibi Sadeer N., Montesano D., Albrizio S., Zengin G., Mahomoodally M.F. (2020). The versatility of antioxidant assays in food science and safety—Chemistry, applications, strengths, and limitations. Antioxidants.

[28] Singh P., Bajpai V., Khandelwal N., Varshney S., Gaikwad A.N., Srivastava M., Singh B., Kumar B. (2021). Determination of bioactive compounds of *Artemisia* spp. plant extracts by LC–MS/MS technique and their in-vitro anti-adipogenic activity screening. J. Pharm. Biomed. Anal..

[29] Olennikov D.N., Chirikova N.K., Kashchenko N.I., Nikolaev V.M., Kim S.-W., Vennos C. (2018). Bioactive phenolics of the genus *Artemisia* (Asteraceae): HPLC-DAD-ESI-TQ-MS/MS profile of the Siberian species and their inhibitory potential against α-amylase and α-glucosidase. Front. Pharmacol..

[30] Mohamed A.E.-H.H., El-Sayed M., Hegazy M.E., Helaly S.E., Esmail A.M., Mohamed N.S. (2010). Chemical constituents and biological activities of *Artemisia herba-alba*. Rec. Nat. Prod..

[31] Melguizo-Melguizo D., Diaz-de-Cerio E., Quirantes-Piné R., Švarc-Gajić J., Segura-Carretero A. (2014). The potential of *Artemisia vulgaris* leaves as a source of antioxidant phenolic compounds. J. Funct. Foods.

[32] Han J., Ye M., Qiao X., Xu M., Wang B.-r., Guo D.-A. (2008). Characterization of phenolic compounds in the Chinese herbal drug *Artemisia annua* by liquid chromatography coupled to electrospray ionization mass spectrometry. J. Pharm. Biomed. Anal..

[33] Bourgou S., Rebey I.B., Mkadmini K., Isoda H., Ksouri R., Ksouri W.M. (2017). LC-ESI-TOF-MS and GC-MS profiling of *Artemisia herba-alba* and evaluation of its bioactive properties. Food Res. Int..

[34] Fabre N., Rustan I., de Hoffmann E., Quetin-Leclercq J. (2001). Determination of flavone, flavonol, and flavanone aglycones by negative ion liquid chromatography electrospray ion trap mass spectrometry. J. Am. Soc. Mass Spectrom..

[35] Han M.H., Park C., Lee D.S., Hong S.H., Choi I.W., Kim G.Y., Choi S.H., Shim J.H., Chae J.I., Yoo Y.H. (2017). Cytoprotective effects of esculetin against oxidative stress are associated with the upregulation of Nrf2-mediated NQO1 expression via the activation of the ERK pathway. Int. J. Mol. Med..

[36] Vasincu A., Luca S.V., Charalambous C., Neophytou C.M., Skalicka-Woźniak K., Miron A. (2022). LC-HRMS/MS phytochemical profiling of Vernonia kotschyana Sch. Bip. ex Walp.: Potential involvement of highly-oxygenated stigmastane-type saponins in cancer cell viability, apoptosis and intracellular ROS production. S. Afr. J. Bot..

[37] Shin M.K., Jeon Y.D., Hong S.H., Kang S.H., Kee J.Y., Jin J.S. (2021). *In Vivo* and *In Vitro* effects of tracheloside on colorectal cancer cell proliferation and metastasis. Antioxidants.

[38] Pruccoli L., Morroni F., Sita G., Hrelia P., Tarozzi A. (2020). Esculetin as a bifunctional antioxidant prevents and counteracts the oxidative stress and neuronal death induced by amyloid protein in SH-SY5Y cells. Antioxidants.

[39] Anand David A.V., Arulmoli R., Parasuraman S. (2016). Overviews of biological importance of quercetin: A bioactive flavonoid. Pharmacogn. Rev..

[40] He J., Xu L., Yang L., Wang X. (2018). Epigallocatechin gallate is the most effective catechin against antioxidant stress *via* hydrogen peroxide and radical scavenging activity. Med. Sci. Monit..

[41] Chang Y., Fan W., Shi H., Feng X., Zhang D., Wang L., Zheng Y., Guo L. (2022). Characterization of phenolics and discovery of α-glucosidase inhibitors in *Artemisia argyi* leaves based on ultra-performance liquid chromatography-tandem mass spectrometry and relevance analysis. J. Pharm. Biomed. Anal..

[42] Carvalho I.S., Cavaco T., Brodelius M. (2011). Phenolic composition and antioxidant capacity of six *Artemisia* species. Ind. Crops Prod..

[43] Xiao J.-Q., Liu W.-Y., Sun H.-p., Li W., Koike K., Kikuchi T., Yamada T., Li D., Feng F., Zhang J. (2019). Bioactivity-based analysis and chemical characterization of hypoglycemic and antioxidant components from *Artemisia argyi*. Bioorg. Chem..

[44] Lomozová Z., Catapano M.C., Hrubša M., Karlíčková J., Macáková K., Kučera R., Mladěnka P. (2021). Chelation of Iron and Copper by Quercetin B-Ring Methyl Metabolites, Isorhamnetin and Tamarixetin, and Their Effect on Metal-Based Fenton Chemistry. J. Agric. Food Chem..

[45] Leopoldini M., Russo N., Chiodo S., Toscano M. (2006). Iron Chelation by the Powerful Antioxidant Flavonoid Quercetin. J. Agric. Food Chem..

[46] Coulanges V., André P., Vidon D.J.M. (1996). Esculetin antagonizes iron-chelating agents and increases the virulence of *Listeria monocytogenes*. Res. Microbiol..

[47] Le Person A., Moncomble A., Cornard J.-P. (2014). The Complexation of AlIII, PbII, and CuII Metal Ions by Esculetin: A Spectroscopic and Theoretical Approach. J. Phys. Chem. A.

[48] Muhammad A., Tel-Cayan G., Öztürk M., Nadeem S., Duru M.E., Anis I., Ng S.W., Shah M.R. (2015). Biologically active flavonoids from *Dodonaea viscosa* and their structure–activity relationships. Ind. Crops Prod..

[49] Nichols E., Szoeke C.E., Vollset S.E., Abbasi N., Abd-Allah F., Abdela J., Aichour M.T.E., Akinyemi R.O., Alahdab F., Asgedom S.W. (2019). Global, regional, and national burden of Alzheimer’s disease and other dementias, 1990–2016: A systematic analysis for the Global Burden of Disease Study 2016. Lancet Neurol..

[50] Lalagkas P.-N., Polyzois S., Papanas N., Nena E., Vourli N., Kontogiorgis C., Constantinides T. (2022). Prevalence of pharmacologically treated type 2 diabetes mellitus in 2012–2016 in Greece: Real-World Data. Prim. Care Diabetes.

[51] Zhang H., Wang Y., Wang Y., Li X., Wang S., Wang Z. (2022). Recent advance on carbamate-based cholinesterase inhibitors as potential multifunctional agents against Alzheimer’s disease. Eur. J. Med. Chem..

[52] Papoutsis K., Zhang J., Bowyer M.C., Brunton N., Gibney E.R., Lyng J. (2021). Fruit, vegetables, and mushrooms for the preparation of extracts with α-amylase and α-glucosidase inhibition properties: A review. Food Chem..

[53] Uddin M.J., Russo D., Rahman M.M., Uddin S.B., Halim M.A., Zidorn C., Milella L. (2021). Anticholinesterase Activity of Eight Medicinal Plant Species: In Vitro and In Silico Studies in the Search for Therapeutic Agents against Alzheimer’s Disease. Evid. Based Complement. Alternat. Med..

[54] Uriarte-Pueyo I., Calvo M. (2011). Flavonoids as Acetylcholinesterase Inhibitors. Curr. Med. Chem..

[55] Mukherjee P.K., Biswas R., Sharma A., Banerjee S., Biswas S., Katiyar C.K. (2018). Validation of medicinal herbs for anti-tyrosinase potential. J. Herb. Med..

[56] Gupta M.K., Senthilkumar S., Chiranjivi A.K., Banik K., Girisa S., Kunnumakkara A.B., Dubey V.K., Rangan L. (2021). Antioxidant, anti-tyrosinase and anti-inflammatory activities of 3, 5-dihydroxy-4′, 7-dimethoxyflavone isolated from the leaves of *Alpinia nigra*. Phytomedicine Plus.

[57] Arroo R.R., Sari S., Barut B., Özel A., Ruparelia K.C., Şöhretoğlu D. (2020). Flavones as tyrosinase inhibitors: Kinetic studies *in vitro* and *in silico*. Phytochem. Anal..

[58] Patel D.K., Patel K. (2022). Therapeutic importance of eriodictyol in the medicine for the treatment of diabetes and associated complication through its insulin secretagogue properties. Metab. Clin. Exp..

[59] Munekata P.E.S., Pateiro M., Rocchetti G., Domínguez R., Rocha J.M., Lorenzo J.M. (2022). Application of metabolomics to decipher the role of bioactive compounds in plant and animal foods. Curr. Opin. Food Sci..

[60] Kaiser H.F. (1961). A note on Guttman’s lower bound for the number of common factors. Br. J. Math. Stat. Psychol..

[61] Czerwińska M.E., Dudek M.K., Pawłowska K.A., Pruś A., Ziaja M., Granica S. (2018). The influence of procyanidins isolated from small-leaved lime flowers (*Tilia cordata* Mill.) on human neutrophils. Fitoterapia.

[62] Luca S.V., Kulinowski Ł., Ciobanu C., Zengin G., Czerwińska M.E., Granica S., Xiao J., Skalicka-Woźniak K., Trifan A. (2022). Phytochemical and multi-biological characterization of two *Cynara scolymus* L. varieties: A glance into their potential large scale cultivation and valorization as bio-functional ingredients. Ind. Crops Prod..

[63] Trifan A., Skalicka-Woźniak K., Granica S., Czerwińska M.E., Kruk A., Marcourt L., Wolfender J.-L., Wolfram E., Esslinger N., Grubelnik A. (2020). *Symphytum officinale* L.: Liquid-liquid chromatography isolation of caffeic acid oligomers and evaluation of their influence on pro-inflammatory cytokine release in LPS-stimulated neutrophils. J. Ethnopharmacol..

[64] Zhang J.-M., An J. (2007). Cytokines, inflammation and pain. Int. Anesthesiol. Clin..

[65] Majdan M., Kiss A.K., Hałasa R., Granica S., Osińska E., Czerwińska M.E. (2020). Inhibition of neutrophil functions and antibacterial effects of tarragon (*Artemisia dracunculus* L.) infusion—phytochemical characterization. Front. Pharmacol..

[66] Ivanescu B., Miron A., Corciova A. (2015). Sesquiterpene lactones from *Artemisia* genus: Biological activities and methods of analysis. J. Anal. Methods Chem..

[67] Svanborg C., Godaly G., Hedlund M. (1999). Cytokine responses during mucosal infections: Role in disease pathogenesis and host defence. Curr. Opin. Microbiol..

[68] de Oliveira Lima M., de Medeiros A.A., Silva K.S., Cardoso G., de Oliveira Lima E., de Oliveira Pereira F. (2017). Investigation of the antifungal potential of linalool against clinical isolates of fluconazole resistant *Trichophyton rubrum*. J. Mycol. Med..

[69] Vega A., Wendel G., Maria A., Pelzer L. (2009). Antimicrobial activity of *Artemisia douglasiana* and dehydroleucodine against *Helicobacter pylori*. J. Ethnopharmacol..

[70] Palacios-Espinosa J.F., Núñez-Aragón P.N., Gomez-Chang E., Linares E., Bye R., Romero I. (2021). Anti-Helicobacter pylori activity of *Artemisia ludoviciana* subsp. *mexicana* and two of its bioactive components, Estafiatin and Eupatilin. Molecules.

[71] Zengin G., Aktumsek A. (2014). Investigation of antioxidant potentials of solvent extracts from different anatomical parts of Asphodeline anatolica E. Tuzlaci: An endemic plant to Turkey. Afr. J. Tradit. Complement. Altern. Med..

[72] Uysal S., Zengin G., Locatelli M., Bahadori M.B., Mocan A., Bellagamba G., De Luca E., Mollica A., Aktumsek A. (2017). Cytotoxic and enzyme inhibitory potential of two *Potentilla* species (*P. speciosa* L. and *P. reptans* Willd.) and their chemical composition. Front. Pharmacol..

[73] Grochowski D.M., Uysal S., Aktumsek A., Granica S., Zengin G., Ceylan R., Locatelli M., Tomczyk M. (2017). *In vitro* enzyme inhibitory properties, antioxidant activities, and phytochemical profile of *Potentilla thuringiaca*. Phytochem. Lett..

[74] European Committee for Antimicrobial Susceptibility Testing (EUCAST) of the European Society of Clinical Microbiology and Infectious Diseases (ESCMID) (2003). Determination of minimum inhibitory concentrations (MICs) of antibacterial agents by broth dilution. Clin. Microbiol. Infect..

[75] Arendrup M.C., Kahlmeter G., Guinea J., Meletiadis J., Testing S.o.A.S.T.o.t.E.E.C.f.A.S. (2021). How to: Perform antifungal susceptibility testing of microconidia-forming dermatophytes following the new reference EUCAST method E. Def 11.0, exemplified by *Trichophyton*. Clin. Microbiol. Infect..

